# Coordinating the Medical Division of Labour: The Travails of Electronic Patient Records in the United Kingdom and United States

**DOI:** 10.1111/1467-9566.70085

**Published:** 2025-09-11

**Authors:** Clare Herrick

**Affiliations:** ^1^ Department of Geography King's College London London UK

**Keywords:** coordination, division of labour, integration, patient records, professions, specialisation, technology, workforce

## Abstract

This paper explores the interrelations between medical specialisation, the changing division of medical labour and the technologies that have emerged to coordinate and integrate patient care. Drawing on the examples of the United Kingdom and the United States, countries whose health systems provide important points of commonality and distinction, I explore the intersections between the rise of medical specialisation and the creation of new medical and paramedical roles. These roles have often emerged as a palliative to the increasing fragmentation and atomisation of medical labour, to ‘assist’ overburdened clinicians and provide better coordinated and integrated patient care. However, as they have proliferated, these new roles have challenged the very nature of work itself. Technology has long held promise as a means of integrating the workforce, service provision and care in ways that might enhance the patient experience and outcomes. Turning to the example of the electronic patient record as one such coordination technology, I explore the travails of its implementation with respect to the healthcare workforce trends and patient care. In so doing, I aim to further contribute to recent scholarship on healthcare workforce trends, role proliferation, ‘taskification’ and strategies to manage the negative externalities of these on patient care.

## 1 Introduction

Over the last century, the absolute size of the medical workforce and the number of its component parts have grown significantly. This has had profound implications for healthcare workers, as well as their patients. As Rosemary Stevens has noted, ‘Medicine, as no other profession, is now functionally fractionated and internally stratified’ (1998, 3). And, although multiple scholars have traced this ‘fractionation’ to the rise of medical specialisation (Weisz 2006; Stevens 1966; Rosen 1972b), the implications of this for broader healthcare workforce trends and patient care have received less attention. Indeed, as this paper explores, the ‘fractioning’, or indeed ‘fracturing’, of medicine through processes including specialisation has necessitated and enabled a proliferation of new healthcare roles and provided an entry point for new services and service providers with consequences for the patient experience and, arguably, health outcomes. Given this, it is instructive that the National Health Service (NHS) now has ‘350 careers’ (NHS England 2025), each of which has many grades, branches, titles and role descriptors, with little consistency between either the four nations of the United Kingdom (UK) or hospital trusts. However, as health services researcher Alison Leary has cautioned, such an expansion of staff and roles has done little to reduce the volume of work being done or to improve patient outcomes. Instead, this insatiable division of labour has served to ‘dilut[e] the workforce by adding more (but usually less qualified) hands, which, in turn, [has] increased the supervisory burden on others and decreased overall productivity and satisfaction levels’ among staff and patients (Nuffield Trust 2023). As this paper will explore, the expansion of the healthcare workforce has itself been both a response to and a cause of the problems of ‘integrating’ and ‘coordinating’ care that have characterised healthcare policy and service delivery reforms for the past six decades (Shaw et al. 2011).

To solve these problems, great hope and significant sums have been invested in new technologies that promise to coordinate and integrate what is increasingly being called ‘taskified’ work, as well as the multiple components of patient care (National Audit Office 2020; Nuffield Trust 2023; Schilling 2025). Here, I use technologies in the Foucauldian sense of techniques or ‘methods and procedures for governing human beings’ (Behrent 2013, 55) that are imbued with power. Thus, rather than the kinds of medical technologies (i.e., X‐rays, stethoscopes etc.) that have often explained the rise of specialisation (Reiser 1978), my concern lies with technologies that corral or coordinate human actions or interactions. Attention to this is important because, although the work of Weisz (2003, 2006), Stevens (1966, 1971, 1998), Rosen (1942, 1972a, 1972b) and others has alluded to problems posed by specialisation and, by extension, its division of labour, their attention has rarely been on attempts to *manage the consequences* of this for patients, healthcare workers or health systems. To consider this, I focus on the example of the electronic patient record (EPR)—also variously called the electronic health record or electronic care record—as an example of a technology that has repeatedly been invested with the hope that it might help reintegrate and re‐coordinate care amid increasing fragmentation.

To this end, I first briefly examine the reasons and rationales behind the turn to specialisation in medicine, with specific reference to the United Kingdom and United States. Second, I explore the imbrication of specialisation with profound shifts in the division of labour within healthcare. Focusing on the creation of new roles, especially those *assisting* or *supporting* doctors or nurses, I argue that as medical labour has been divided, atomised and, in the process, often reduced to ‘tasks’, it has become less legible to patients and frequently harder to navigate. Third, I turn to the example of EPRs as a technology that is often marketed as capable of better integrating and coordinating not just patient care, but also clinical workflows and decision‐making. Drawing on detailed analysis of coverage of EPRs in the *British Medical Journal* and the *Journal of the American Medical Association* from 1990 onwards, I explore how the ‘shared theory of improvement’ invested in the EPR (Vikkelsø 2005, 4) has emerged as an aspirational antidote to some of the problems of fragmentation: between different specialisations; hospital, primary and community care settings; and different role holders. However, the seemingly simple ambition to create a unified patient record legible across multiple providers has been beset with problems on both sides of the Atlantic. The limits of EPRs are worthy of further analytical scrutiny within the context of the medical division of labour because the EPR cannot be understood simply as ‘a solution to poor coordination or continuity’ (Sheaff et al. 2017, 1030), but also as a technology that itself is generative of new problems for both healthcare providers and patients.

## 2 Specialisation: (Dis)integrating Medicine

One of medicine’s greatest triumphs, as well as its most significant failings, has been the rise of specialisation since the mid‐19th century. In his classic text, George Rosen views specialisation as a process whereby the ‘total field of practice’ becomes split into a ‘number of sub‐fields or specialties’ and each of these ‘comes to occupy the attention of a certain group of physicians known as specialists’ (1972, 3). Specialities, as a ‘focus of interest’ and a ‘field of practice’ (1972, 4), are created through processes of ‘segmentation’ or ‘accretion’ (between two fields). They are, he argues, generally oriented around three domains: technical procedures (i.e., radiology); organs or organ systems (i.e., nephrology); or age groups (i.e., gerontology or paediatrics). Specialisation itself was facilitated through the concept of ‘localised pathology’, which enabled organs or organ systems to become new ‘foci of interest’ for clinicians. This, in turn, provided the conditions of possibility for new technologies, investigative and diagnostic methods, and expertise (1972, 10). Such increasing precision of professional focus has been described by some as a form of ‘atomisation’ (Epstein and Back 2015) that undermines the ability of medicine (and healthcare more broadly) to address the ‘whole person’ (D. Armstrong 1986). Thus, although specialisation has doubtlessly propelled innumerable medical advances and improvements in patient care and outcomes, it has also been accompanied by negative externalities that have required active management.

Stevens (1998) argues that specialisation was as much an outcome of professional politics and the imperfect workings of the medical labour market as it was advances in medical technologies or changing modes of diagnosis. For specialists, the economic rewards of specialisation compared with the relative grind of general practice provided (and continue to provide) further incentives, as did the social prestige that technical expertise conferred. Although the United Kingdom was far slower to accept and promote medical specialisation than the United States, Weisz (2003) notes that a growing cadre of specialists and a proliferation of international scientific meetings helped propel interest in speciality fields and techniques. Still, as he argues, by the time of the advent of the NHS in 1948, there remained a ‘uniquely small number’ of specialists (2003, 575), and therefore a profound need to increase their numbers to satisfy the demands of the new service (2006, 182). This need has, arguably, never been satisfied (see e.g., Britnell 2011). Specialisation has, at least in the United States, tended to be a route to medical riches, and in a thoroughly imperfect market such as healthcare, the result has been a maldistribution of specialist care that reflects market opportunity far more than patient need (Rosenthal et al. 2005). This has further deepened the fragmentation of care itself and the challenges of coordinating disparate providers, especially in the context of multimorbidity or complex, chronic conditions.

To think through the challenges of coordination posed by these fragmentary forces, some sense of the scale and scope of change is helpful. Rosen’s (1972a, 1972b) history of medical specialisation contains an anecdote that illustrates just how quickly medical roles proliferated as specialisation took off: The notes of an American man with heart disease showed that in 1908 he was attended by six people, including two doctors. In 1938, the same condition warranted the attention of 32 people, including no fewer than 10 specialists. Reiser (1978) evidences a similar trajectory: In 1929, 25% of all doctors declared themselves to be specialists; by 1969, it was 75% (ibid.). The corollary of this turn to specialisation in the United States was a precipitous fall in the number of general practitioners (GPs)—now the speciality of primary care or family medicine—from 21% of all doctors in 1968 (Stevens 1971) to roughly 12% now (Association of American Medical Colleges 2021). The American Board of Medical Specialities (2024) now lists 38 specialities and 89 subspecialities. And, from its inaugural four ‘member boards’ in 1933 (dermatology, obstetrics and gynaecology, ophthalmology and otolaryngology), it now has 24. These shifts have not only changed the American medical but have also profoundly shaped all aspects of healthcare.

In the United Kingdom, the past five decades have witnessed a similar turn to specialisation, super‐specialisation and sub‐specialisation. This has, however, occurred at a scale and scope that are far more limited given the continued centrality of general practice to routine medical care and as the gatekeeper to specialist care. In the United Kingdom, specialisation is thus not the dominant form of medical practice that it has become elsewhere. For example, the UK’s General Medical Council lists 65 specialities and 31 subspecialities whose curricula are designed by the relevant royal colleges and faculties. Consultants, however, make up only 4% of England’s NHS workforce, and this *proportion* has stayed roughly the same for the past decade (Moberly 2017a). In the United States, an over‐proliferation of specialists in many parts of the country has produced inefficiencies and cost inflation associated with a lack of access to and use of primary care. By contrast, in the United Kingdom, patients grapple with a lack of timely access to consultant‐led care once they have been referred by a GP. This is despite consultant *numbers* doubling and the proportion of the NHS budget spent on hospitals increasing from 47% in 2010 to 56% in 2024 (Darzi 2024).

In workforce terms, specialisation has often been described as having an ‘atomising’ effect that has necessitated new working arrangements such as ‘multidisciplinary teams’ or ‘integrated care systems’ (Whitfield and Schlich 2015). The extent of this atomisation is arguably far greater in the United States where the American Medical Association’s Physician Masterfile extends to 48 categories of specialisation (Association of American Medical Colleges 2021), compared to the 12 listed in the NHS workforce statistics. Indeed, an American medical student can now choose from a bewildering list of 189 specialities for their residencies, including no fewer than nine radiology specialisms and 22 surgery specialisms (including three separate categories of hand surgery) (American Medical Association 2024). This plethora of choice obviously has profound implications for medical careers, but it also has an impact on the equitable provision of healthcare services, patient access and the continuity of care. Put another way, what does it mean for patient experience and outcomes and the provision of services when there are 189 specialisms? One way of approaching this question is to consider the changing division of labour and workforce trends that have aimed to mitigate the effects of fragmentation on patients and healthcare workers themselves, as well as to provide new forms of integration and coordination between people and services.

## 3 ‘Taskification’, the Changing Division of Labour and the Case for Integration

The 1960s and 70s were a period of intense concern with the impact of specialisation on patient care and outcomes, as well as the escalating costs of medical care in the United States (Stimmel 1975). This was afforded even greater urgency by the advent of Medicare and Medicaid in 1965, which not only shifted the social contract between the state and patients in relation to the right to health services but also transformed healthcare into an industrial behemoth directly supported by federal subsidy (Stevens and Stevens 1974; Relman 1980). Around the same time, the ‘limits to’ medicine (Illich 1974) and the ‘de‐humanisation’ of medical care (Howard et al. 1977) emerged as a source of anxiety, as individual clinicians attended to specific aspects of disease and the needs of the whole patient were often lost (Paik 2000). Patient concerns over quality of care and equity of provision have doubtlessly been fuelled by the fact that ‘There has been no one master strategy for the development of a medical profession stratified into specialties’ (Stevens 1971, 10). Because of this, mechanisms that enable the ‘control and guidance’ of specialist practice and, therefore, the management of its implications for patients and the healthcare workforce have become essential (Rosen 1972a). This has especially been the case in the United States, where the coordinating (triage, referral, follow‐on etc.) role of the GP is far less common. And, when ‘numerous independent specialists treat the same patient’, the result has often been that any ‘improvements in care and reductions in cost resulting from having more highly trained specialists deliver specific services [have been] offset by the quality‐eroding and cost‐increasing effects of the multiple communications required’ between these different providers (Detsky et al. 2012, 463). Although the American medical profession may be ‘an abnormal form of the division of labour’ (Fryer 1991, 213) in the Durkheimian sense of lacking any form of inter‐professional ‘solidarity’, it is also a rational response to a lack of any overarching attempts to plan what form and distribution of specialisation might be optimal at a systemic level in the delivery of care (Baumgartner 1971).

One of the early solutions to the American problem of over‐specialisation, the relative decline of general practice and family medicine (now commonly referred to as primary care) and the ‘demographic rigidity’ of medicine's career and training paths (Abbott 2014, 130), was the creation of new roles to shift tasks deemed to be ‘simple’ away from doctors. As Eliot Freidson remarks, ‘The natural history of the so‐called helping professions in [the United States] has very consistently been one in which low‐status occupations were invented and trained for the performance of tasks that higher‐status occupations no longer wished to perform’ (1975, 225). One of the most well known of these low(er)‐status professions is the physician associate (initially called the physician assistant) introduced at Duke University in the late 1960s (Stead 1966). The early rationale for (and promotion of) this new dependent, ancillary role was to address workforce shortages, ‘free up’ doctors to perform complex clinical work and, in the process, improve the coherence and quality of patient care and health outcomes (Freilich 1975; Sadler et al. 1972). As one of a ‘host of subordinate groups under medicine's dominion’ (Abbott 2014, 72), the physician associate role has since expanded into hospital care, assisting specialists as much as generalists. Their job descriptions may have changed, but the role's justificatory narrative of improved and better integrated patient care remains. A 1999 ‘Strategic Plan for the Physician Associate Profession’ makes this clear: ‘They work in complementary and synergistic ways with physicians. They foster an *integrated* rather than a *fragmented* care system, assure continuity of care and embrace the tenet that two heads are better than one’ (Editors 1999, 76).

Just as the need for better integration amid fragmentation helped justify the need for physician associates from the late 1960s, the 1978 Royal Commission on the NHS similarly recognised that the profession needed to adopt more ‘flexible staffing structures’ to accommodate the ‘continuous although unstructured process in which tasks and functions are redistributed between professions’ as demands on the healthcare system evolved (Merrison 1979). Although the extent of specialisation in the United Kingdom has never reached that of the United States, the question of *who* should be charged with *which tasks* within a health service increasingly characterised by an ever‐increasing division of labour has persisted. As a result, and because of resource constraints, ‘Aides and unqualified staff should be used where possible, releasing skilled workers for jobs that require their expertise’ (ibid., n.p.). As Abbott has noted, the creation of new roles is a force with unstoppable momentum largely because ‘subordinates become absolutely necessary to successful practice by superordinates’ (2014, 72). The strict hierarchies, training, competencies, accreditation, licensing and regulation of the healthcare professions mean that fragmentation is both endemic and essential to the functioning of the system itself. However, for patients, an increasingly fragmented labour force can be hard to understand, difficult to navigate and may not always be a pathway to better care (see e.g., Gentry 2021; Roberts et al. 2023).

**FIGURE 1:**
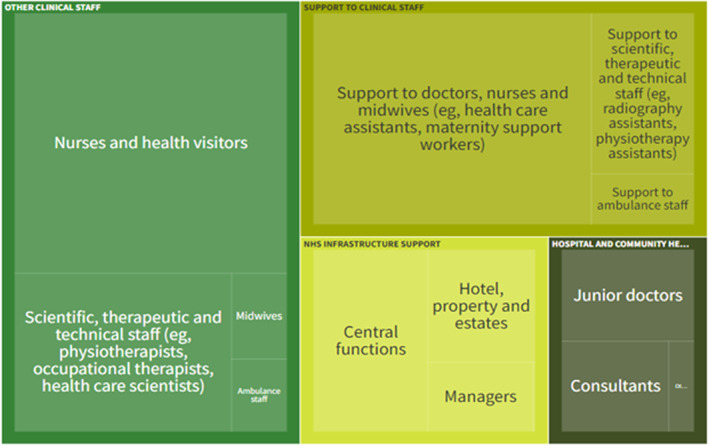
*Source:* NHS workforce statistics (Mallorie 2024).

From physician associates to advanced nurse practitioners (a role within which there are now 27 specific titles, including family nurse practitioner and clinical nurse specialist), the expansion of roles and introduction of new ones has changed the nature of healthcare provision. Yet, the creation of new, dependent roles may not always be additive. Instead, it may complicate and fragment the division of labour further as tasks need to be delineated and assigned to role holders according to their competencies and scope of practice. To give an example, the United Kingdom had 138,604 doctors and 363,226 nurses, midwives and health visitors in 2023. By comparison, it had 405,089 people working in roles that offer ‘*support* for clinical staff’ (Office for National Statistics 2024). These figures point to a rapidly changing (and arguably poorly understood) division of labour (Palmer et al. 2025), with an ever‐greater proportion of the workforce now *supporting* clinical and ‘scientific, therapeutic and technical’ staff (see Figure 1). Efforts to address workforce shortages, particularly in general practice, have also fuelled this trend. For example, the NHS has invested heavily in paramedical support, as well as aptly named ‘link’ and ‘navigator’ roles through schemes such as the Additional Roles Reimbursement Scheme (ARRS) (MacConnachie 2024). Introducing these has, in some cases, helped improve access to primary care through reduced waits for appointments (Greenhalgh and McKee 2025), but evidencing that they have improved patient outcomes is marred by inconsistent definitions and deployment of the role itself (Tierney et al. 2019). New paramedical roles have, however, increased the supervisory burden on qualified GPs and registered nurses, creating more tasks for a group whose absolute numbers have not increased. The expansion of roles has thus altered the topography of healthcare provision and, in the process, created new challenges of coordination for both staff and patients.

Although the composition of the NHS workforce is shown in Figure 1, the categories shown belie the huge number of ‘roles’ and job titles that exist within each. There are an estimated 77,000 different job titles in the English NHS (Collins 2023), the sheer volume of which suggests that an uncoordinated atomisation of labour is underway. To use the example of nursing, the 595 job titles currently in use across the NHS often hold little logical connection to educational pathways, qualifications, years in post, registration or regulation (Leary et al. 2017). Moreover, as job titles and descriptions are often a creation of individual employers (i.e., NHS trusts) rather than a function of regulatory or accrediting bodies, such variation creates confusion around role function, clarity, professional identity and commensurability across healthcare settings. This confusion extends to colleagues as much as patients. Maxwell and Leary have bemoaned the tendency of workforce planning to work from the Taylorist premise that

The division of labour should be based primarily on technical competence; that is, a worker’s capability to complete a series of independent tasks of varying complexity in a standardised manner. A small number of decision makers determine the tasks that need to be completed through the development of guidelines, checklists, and protocols. The workforce is then assessed against a set of task competencies, but never against the need to balance competing tasks, or to consider the unintended consequences of one task on another aspect of the workload (2020, n.p.)

The result, they argue, is that professional practice has been reduced to ‘task delivery’. This format is ill‐suited to the rising prevalence of complex, chronic co‐morbidities, which requires the practical coordination (and oversight) of care across different specialisms, services and settings. This presents a challenge to existing workforce structures that have not necessarily kept pace with the new ways of working demanded by such epidemiological shifts. In the NHS at least, ‘success’ is increasingly ‘measured by *how much* work is done, not *how well* work is done’ (Leary 2023, n.p., emphasis added). As a result, new roles have been created to attend to the ever‐increasing ‘volume’ of work that also serves as its own metric of success. In a later talk for the Nuffield Trust, Leary termed this ‘tick‐box approach’ to healthcare ‘taskification’ (2023), an idea taken up in a recent BMJ opinion piece arguing that ‘patients can now travel through health systems without ever meeting or needing a doctor’ (Morgan 2024, 155). This seemingly counterintuitive situation—especially at a time of growing doctor numbers—comes about because medicine itself is being ‘taskified’ into ‘discrete events’ which are ‘delivered through a multitude of roles’ in a health system that increasingly resembles a ‘factory line’ (ibid.). Such ‘taskified’ work may originate in medical education itself, with ‘competency‐based medical training’ broken down into ‘units of professional practice’ and the ‘bureaucratic exercise’ of ‘assessment events’ (Strand et al. 2025). Back in medical practice, within this new division of labour, doctors must supervise ever more people who ‘support’ or ‘assist’ them but who are not permitted to ‘substitute’ for them. This often leaves them with *less* time to do their own work with attendant effects on patient care (Greenhalgh and McKee 2025).

As numbers of clinical, technical and support staff have mushroomed, new technologies of coordination have consequently become essential to reintegrate not just tasks and people, but also clinical and community *care* (Berg 1998; Timmermans et al. 1998). However, doing so requires a shared understanding of what, exactly, integration and coordination are, how they might be operationalised and to what effect. The solution to a lack of integration has, unsurprisingly given the fragmentary trends discussed, been new fields: the ‘multidisciplinary care’ of the 1960s, the ‘partnership working’ and ‘health team’ of the 1970s, ‘coordinated care’ in the 1980s, ‘managed care’ in the 1990s and ‘integrated care’ in the most recent decades (Shaw et al. 2011). Although the nomenclature may have shifted, the operational ambition to address the problems of ‘a lack of service coordination for individual patients’, the ‘structural and cultural isolation of generalist from specialist medicine, or adult social care from health care’ and the resulting ‘discontinuity of care’ (ibid., 7) has remained fairly constant, at least in the United Kingdom. And yet, the aspiration of integration—or indeed coordination—is marred by the same complexities that have characterised the explosion of healthcare roles. With at least 175 definitions and concepts of ‘integrated care’ alone (Armitage et al. 2009) and sparse evidence to suggest that integrated care, however defined or operationalised, actually leads to improved patient outcomes (Hughes et al. 2020), it is easy to see why some technologies have become posed as ‘universal’ solutions to endemic (dis)integration (Sheaff et al. 2017). As the remainder of this paper will explore, coordination has become the means by which better healthcare integration is made possible (Shaw et al. 2011). To achieve this, technologies such as the EPR offer ‘coordinating functions’ that can be layered over different modes, structures and processes of healthcare delivery (Poku et al. 2019).

## 4 Technologies of Coordination: The Travails of the Electronic Patient Record

One of the main casualties of increased specialisation and increasingly atomised divisions of labour has been the coordination of patient care (Stange 2009). Patient care pathways, particularly for chronic conditions, are often extended out to multiple providers, teams and locations. With little clarity over who *coordinates* these services, new technologies (and roles) have emerged to ‘navigate’ the patient experience, increase efficiencies and, ideally, improve patient outcomes. In some cases, this has meant a reorientation of specialisms themselves, with geriatric and hospital medicine in the United States underpinned by a fundamental ethic and practice of coordinated care. On both sides of the Atlantic, the EPR has emerged as a theoretically simple—but practically far more complex—means of achieving the ‘interorganisational coordination’ (Vikkelsø 2005, 4) necessary to overcome the problems of fragmentation (Brennan 2001).

Inevitably, the framing of this technology as both solution (and itself a problem) has often been quite different in the United Kingdom and United States, despite both countries sharing similar concerns over the health consequences of fragmented patient care. The medical record serves a dual role—both reflective of the clinical encounter and feeding into it—meaning that any change in form, function or process alters both. As Berg notes, the medical record ‘structures the communication between healthcare personnel, shapes medical decision‐making and frames relations between personnel and patients’ (1998, 297). At its most basic functionality, the EPR should allow multiple healthcare role holders to share information about ‘all care events on a patient's journey’, including their medical history, upcoming and past appointments with multiple providers, treatment plans, medications, allergies and adverse reactions and, as a result, to be able to offer more efficient, safe, consistent and integrated care (Lawrence et al. 2025). As such, it becomes a tool of care coordination in that it offers one constant point that can travel across the complex topography of healthcare provision that this paper has explored.

To make this aspiration a reality, the NHS ran a ‘clinical terms’ project in 1992 that sought to generate a common language across the health service. The idea was that this would be the basis of the interoperable IT systems needed to bridge general practice and hospital settings to ensure that patient care could be integrated between the two. This was less of an issue for GPs who had been strongly incentivised to adopt computing systems for all aspects of service provision and patient record keeping since the 1970s. In hospitals, however, IT systems were often seen as a time drag with little clear benefit by consultants. This gap in uptake partly drove the 1998 *Information for Health* strategy and the subsequent 2002 *National Programme for Information Technology*. By this point, almost all GP practices were 100% digital (Department of Health and Social Care 2016), but hospitals still lagged behind. The strategy set a target to introduce EPRs at a third of all acute hospitals containing four sets of information: prescribing details, tests ordered, results and the ‘integrated care pathway’ that was being used (Burke 2002). In practice, this clearly delimited set of domains points to the operational limits of the aspiration of seamless, coordinated care touted by technology providers and dreamt of by patients (Sheaff et al. 2017).

Even in the NHS, hospital and GP systems, services and clinical workflows are so wildly different that developing a bespoke IT system able to bridge the two was quickly discovered to be virtually impossible. As Benson notes, EPRs ‘serve an enormous range of tasks, including direct patient care, preventive care, clinical decision support, audit and accountability, legal evidence, management and financial control, clinical trials, research, and comparisons (local, national, and international)’ (2002, 1090). EPRs consequently function best where the scope of use is clearly delineated. This is narrowest within general practice, where most patient encounters are one‐to‐one, and system functions are more circumscribed. By contrast, ‘Hospital medicine has complex workflow, job specialisation, and division of labour, which creates complex and diverse patterns of information use…the mode of information use is different in intensive care, on inpatient wards, at outpatient clinics… with regard to variables such as the volume and half‐life of data, the need for rapid response, and the value of decision support tools and integration with medical devices’ (Benson 2002, 1090). For this reason, EPRs have remained both a necessity (to overcome workforce fragmentation) and a challenge (because of fragmentation) in hospitals on both sides of the Atlantic.

This initial failure of the NHS hospital EPR was more than two decades ago, and problems have stubbornly persisted. In 2002, Derek Wanless declared the service's systems as ‘piecemeal and poorly integrated’ (Department of Health 2002). A new target was set for all acute NHS trusts to have EPRs by 2007 with three components: prescriptions; ‘choose and book’; and a lifelong health record ‘spine’ (Cross 2006). This was again beset with issues including staff morale, skills and a lack of inclusion of clinicians in systems design to ensure its ‘usability’ (Hendy et al. 2005). Around this time, patient confidentiality concerns emerged because the ‘spine’ of the EPR (by this point called the ‘summary care record’) would be accessible to a multitude of users. Although this was viewed by civil liberties campaigners as an infringement of privacy (Norheim 2006), it was precisely what was needed to improve integration and coordination. Consequently, when the BMA called on its members not to cooperate with the EPR rollout due to security and confidentiality concerns in 2007 (Gornall 2008), this placed it at odds with the many patient groups arguing that EPRs were essential for better coordinated care across a fragmented workforce (Cross 2011a; O’Dowd 2010). In 2011, the government announced the end, for the second time, of the national IT plan (Cross 2011b). And in 2012, the King's Fund and Nuffield Trust issued renewed calls for better integrated care, central to which would *again* be the EPR, among other technological innovations (Hawkes 2012).

At this time, criticism of the NHS's ‘luddite approach’ to IT services grew, with the lack of any integrated system making the lives of junior doctors even harder (Moberly 2014). In 2017, a new target was set for a ‘paperless NHS’ by 2020 (S. Armstrong 2017; NHS England 2017). This time, the potential benefits for the efficiency of clinical services—for both doctors and patients—were more forcefully made (Moberly 2017b). Combined with the digital advances propelled by COVID‐19, EPRs have become more common, although not yet ubiquitous (Lawrence et al. 2025; National Audit Office 2020). Indeed, some have argued that EPRs and their use remain as fragmented as the patient care they were originally intended to integrate (Chan et al. 2023). With EPRs often ‘touted as enabling all staff to work from a single record, presenting a clear overview of a patient’ (Best 2023, 5), the reality remains that EPR systems from different trusts may not be legible or accessible to another even as patients have to navigate numerous healthcare settings to access services. Many staff are consequently still ‘wrangling with fragmented electronic systems that are clunky, counterintuitive and not always compatible’ (Oliver 2023, 1). These do nothing to lessen the bureaucratic work undertaken by clinicians and may pose potential safety risks to patients should their vital information not end up being accurately shared across different healthcare settings.

Turning to the United States, the travails of EPRs speak clearly to the hope placed on the technology to ‘seamlessly’ integrate and coordinate an intensely divided medical system in the name of patient outcomes (Vikkelsø 2005, 4). The early justification (often pushed by the technology providers themselves) for the introduction of EPRs was linked to patient choice, as well as improving clinical practice and patient safety (Safran 2001). Importantly, in a familiar narrative that sits in contrast to the United Kingdom, EPRs were actively sold to doctors as a labour‐saving tool that would ‘free up’ their time for better quality clinical encounters and enhanced collaboration with colleagues. The technology was heralded as essential to improving population‐based health research and interventions, especially as EPRs could theoretically be used to group patients by risk factors (Rashbass 2001). Ultimately, it was hoped that this would enable improved and evidence‐based clinical decision‐making (in the absence of the kind of guidance used in the United Kingdom through the National Institute for Health and Care Excellence). For large US healthcare providers, the potential of EPRs as a billing, administrative and (financial and clinical) audit tool was also a significant driver for their uptake, as well as providing the narrative bedrock of the sales pitch used by technology companies to sell their wares. Indeed, as US provider Epic claims, the technology can ‘modernise your health system's workflows and optimise your resources’ with a ‘robust functionality that transforms patient experiences and satisfies physicians’ (Core International 2025).

A turning point for EPRs came in 2009 when President Obama promised to digitise the health records of all Americans within five years (Greenhalgh 2009). The President's American Recovery and Reinvestment Act offered $19 billion in incentives for the adoption and ‘meaningful use’ of EPRs and a further $50 billion in incentives to promote healthcare IT (D’Avolio 2009). The Health Information Technology for Economic and Clinical Health (HITECH) Act of 2009 was the mechanism by which this shift was enacted at a national scale. However, the American healthcare ‘system’, which Grumbach describes as ‘extravagantly rococo’ (2009, 2363) in its structure, has never been the ideal environment within which to aspire to the universal uptake of EPRs. The HITECH Act set a high bar for the ‘meaningful use’ of EPRs, ensuring that just *having* the technology was not sufficient to avoid federal reimbursement penalties (Jha 2010, 1709). And although the EPR made the integration and coordination of care theoretically feasible, the degree of transformation promised by technology providers has remained limited by the overarching structural problems of the US healthcare system itself.

In recent years, the NHS has managed to achieve some of its IT goals, but a lack of resourcing (especially in operational research, quality improvement, data science and clinical informatics teams) means that they are not currently being used to their full potential (Lawrence et al. 2025). The United States remains home to a patchwork of systems that are not always interoperable and are increasingly the subject of vehement critique for their contribution to staff burnout, stress and a lack of time with individual patients. This problem is magnified by the fact that EPRs often work against, rather than with, clinical workflows, especially with regard to patient communication and consultation (see also Berger et al. 2024; Cutler 2019, 1963). They also, as a study in Denmark has shown, can reconfigure the practice of healthcare itself, creating new tasks, eradicating others, shifting workloads and re‐allocating tasks to new professional groups (Vikkelsø 2005). To return to the division of labour, the proposed solution to the added bureaucratic burden of the EPR has been the introduction of *new* clinical and technical roles or the addition of new *tasks* to old roles. One that may be familiar to historians is the medical scribe, now touted as a novel ‘clinical adjunct’ that can reduce the ‘administrative burden’ of clinicians newly bound to EPRs by ‘freeing up’ their time so that they can focus on the ‘patient‐doctor experience’ instead of writing up notes (Nambudiri et al. 2018). Other roles include, for example, ‘patient flow leads’, ‘care coordinators‘, ‘services advisors’ or ‘coordination administrators’, all of whom, in various ways, seek to manage the movement of patients through and across different care settings, with the EPR an essential tool needed to facilitate this work. Recent studies have also shown that the use of EPRs by doctors has tended to *increase* personnel requirements, with American doctors more likely to devolve the task of writing up patient notes, ordering tests or prescriptions to a physician associate for the sake of ‘efficiency’ (Berger et al. 2024).

As the uptake of EPRs has climbed, the backlash against them has grown, and ‘as currently configured, some contend that [it] actually makes caring for patients much more difficult’ (Sanders et al. 2020, 34). The capacity of the EPR to erase the deep connection between patient and doctor by inserting the distraction of the digital has also become a worry. As Toll notes, ‘Not surprisingly, we find ourselves entering more and more data while we are trying to listen to and talk with our patients’ (2020, 1661). This ‘intrusion into the clinical encounter’ (Detmer and Gettinger 2023, 1825) has been cited as one cause of declining patient satisfaction with some medical services (Toll 2020, 1662). As Reiser noted, ‘Despite [its] unifying role, caution is needed in the face of excessive expectations about how transformative the [EPR] can be’ (2009, 103). In the United Kingdom, this caution has largely been about the cost of the IT rollout to the NHS, staff training, residual interoperability issues and patient confidentiality. In the United States, the EPR has frequently been viewed as another bureaucratic tool that promises more than it delivers within a healthcare system so fragmented as to make integration systemically impossible. And yet, even in a centralised system like the NHS, the EPR has still largely failed to provide the coordination needed to offer a fully workable solution to fragmented care.

## 5 Conclusion

As I have argued and explored in this paper, the fragmentation of healthcare has necessitated new forms of technology to coordinate and reintegrate its disparate components into a more functional and legible system for healthcare workers and patients. As such, the EPR has long been constructed as a tool ‘capable of generating [the] future’ of healthcare (Jensen 2005, 255). For the last 30 years, EPRs have also been consistently imbued with hope and frustration. These emotions have taken different forms in the United States and the United Kingdom, as would be expected given the significant differences in their healthcare systems, professional composition of their health workforce and cultures of medicine. However, the work to introduce and mainstream EPRs across both countries illustrates not only the shared problems that the increasing atomisation of the medical division of labour poses, but also how this seems to be a largely unstoppable force. The fact of EPRs helping coordinate some aspects of care (e.g., prescribing or appointment booking) does not obscure the fact that the new technology has inevitably created new problems to be managed. These include the following: changes in clinician workflow; the increased burden on already overstretched clinical staff (Walsh 2004); the call for even more new roles to manage and analyse EPRs; and most recently, concerns over the concentration of this vital infrastructure in the hands of just a few software companies. For example, Epic is estimated to hold a 35% market share of EPRs in the United States (Joseph 2024), whereas freedom of information requests to NHS trusts in the United Kingdom show the same company is the EPR provider for 13 out of 214 trusts (6%), with Rio for 12% and Cerner for 16% of trusts (6b Digital 2025). With 10 major EPR providers in the United Kingdom, the technology may be less monopolised than in the United States but risks remain.

The example of EPRs thus lays bare the fundamental challenge of coordination in healthcare systems that are increasingly fragmented and where some roles are necessarily being reduced to the execution of ‘tasks’ rather than ‘care’. As the ‘skills mix’ shifts and a greater proportion of the workforce occupies ‘supporting’ roles, integration becomes more challenging just as it becomes more essential (Palmer et al. 2025). It is worth recalling that when physician associates were introduced in the United States six decades ago, the new role was sold to doctors as a way of ‘freeing up’ their time from the ‘simple tasks’ that an assistant of lesser training could be entrusted to undertake under their supervision (Herrick 2025). At the same time, the role was sold to patients as a means of getting better access to more integrated care. This demonstrates how the shift to specialisation (and thus away from generalisation) not only necessitated new divisions of labour to deliver adequate patient care but also rendered (dis)integration a problem to be overcome. Little has arguably changed, with the NHS similarly and belatedly introducing new ‘task shifting’ roles that principally aim to fill staffing gaps far more quickly than would be possible with fully trained doctors or nurses (National Audit Office 2024). These have, however, created new frictions over the division of labour and its effects on service delivery and patient care. The recent debates over ‘scope of practice’, level of (in)dependence and whether roles are an ‘assistant to’ or ‘substitute for’ doctors and nurses that reached fever pitch among NHS staff, politicians and academics in 2024 (Abbasi 2024; Greenhalgh and McKee 2025) demonstrate these frictions in practice. And, in a taxpayer‐funded healthcare system, these are not merely esoteric workforce concerns but represent crucial questions about what forms of labour are valued and worthy and can deliver the services and care that patients need and expect.

As a final reflection, healthcare systems, wherever they may be, trend inexorably towards expansion (Baumol 2012). In both the United States and the United Kingdom, the size of the healthcare workforce has grown significantly over time and now stands at 17 million people in the former (National Center for Health Workforce Analysis 2024) and 1.7 million in the latter (Mallorie 2024), making it the largest employment sector in both. Looking forward, the NHS health workforce is predicted to grow by a further 65% to 2.3 million by 2036 (National Audit Office 2024), although significant assumptions about workforce substitution have been built into these projections. Similarly, in the United States, although the number of doctors is projected to grow by 4% over the next decade, newer paramedical and support roles are set to increase at far higher rates (US Bureau of Labor Statistics 2024). Although this process would seem unstoppable, the recent vote to phase out the physician associate role within UK general practice (Royal College of General Practitioners 2024) demonstrates the capacity to push back on new roles given their tendencies to exacerbate the problems of fragmentation, especially where patient safety is compromised. Arguably then, the interlinkages between medical specialisation, new forms of the division of labour and attempts to integrate through technologies of coordination such as the EPR point to the importance of analysing and understanding the workforce trends explored in this paper. And, in healthcare, these have a life‐or‐death salience that extends far beyond job titles and role descriptors.

## Author Contributions

**Clare Herrick:** conceptualization, investigation, writing – original draft, methodology, writing – review and editing.

## Conflicts of Interest

The author declares no conflicts of interest.

## Data Availability

Data sharing is not applicable to this article as no datasets were generated or analysed during this study.
